# Nocturnal pollination: an overlooked ecosystem service vulnerable to environmental change

**DOI:** 10.1042/ETLS20190134

**Published:** 2020-06-01

**Authors:** Callum J. Macgregor, Alison S. Scott-Brown

**Affiliations:** 1 Leverhulme Centre for Anthropocene Biodiversity, University of York, York YO10 5DD, U.K.; 2 Energy and Environment Institute, University of Hull, Cottingham Road, Hull HU6 7RX, U.K.; 3 Royal Botanic Gardens Kew, Richmond TW9 3AB, U.K.

**Keywords:** artificial light at night, biodiversity, climate change, plant-insect interactions, pollinators, pollution

## Abstract

Existing assessments of the ecosystem service of pollination have been largely restricted to diurnal insects, with a particular focus on generalist foragers such as wild and honey bees. As knowledge of how these plant-pollinator systems function, their relevance to food security and biodiversity, and the fragility of these mutually beneficial interactions increases, attention is diverting to other, less well-studied pollinator groups. One such group are those that forage at night. In this review, we document evidence that nocturnal species are providers of pollination services (including pollination of economically valuable and culturally important crops, as well as wild plants of conservation concern), but highlight how little is known about the scale of such services. We discuss the primary mechanisms involved in night-time communication between plants and insect pollen-vectors, including floral scent, visual cues (and associated specialized visual systems), and thermogenic sensitivity (associated with thermogenic flowers). We highlight that these mechanisms are vulnerable to direct and indirect disruption by a range of anthropogenic drivers of environmental change, including air and soil pollution, artificial light at night, and climate change. Lastly, we highlight a number of directions for future research that will be important if nocturnal pollination services are to be fully understood and ultimately conserved.

## Introduction

The close co-evolution of plants and pollinators has fascinated biologists for well over a century, evident by the extent and depth to which key pollinating species and their flower-visiting interactions have been investigated and reported [1]. The ability of plants to attract, reward and exploit effective pollinators is key in the diversification of floral traits to maximize reproductive success and maintain gene flow [2]. Nocturnal pollination is arguably one of the most intriguing facets of this discipline, yet it remains little explored. This is surprising as plants require sophisticated adaptations to ensure that floral signals are detectable to nocturnal pollen-vectors [3]. There are numerous benefits for nocturnal functioning for both partner organisms. For example, pollinators are able to feed in relative safety in absence of diurnal predators, while avoiding direct competition from most bees for pollen and nectar [4]. Plant partners can reduce their visibility to foraging antagonists, while increasing pollination efficiency and/or reducing the risk of heterospecific pollen interference (given that generalist diurnal pollinators, e.g. bees, may visit other sympatric flowering plants over the course of their lifetime [5]) [6]. Additionally, recent work suggests that many generalist flowers are visited nocturnally as well as during the day [7]. Despite these facts, nocturnal pollinators have been less frequently studied (Table 1), and may therefore be undervalued.

**Table 1:** Nocturnal pollinator taxa, ordered by hits to searches in Web of Science and Scopus

Taxon	Search terms		Search engine hits		Web of Science hits with additional search terms:			
	Generic	Taxon-specific	Web of Science	Scopus	AND (‘ecosystem service*' OR ‘pollination service*')	AND (‘floral scent' OR ‘floral volatile*')	AND (‘climate change' OR ‘climate warming' OR ‘global warming')	AND (‘artificial light at night' OR ‘light pollution')
**Nocturnal pollinator taxa**	pollinat* AND (nocturnal OR night) AND…							
Bees		(Hymenoptera OR Apoidea OR bee*) NOT ant	385	380	11 (2.9%)	61 (15.9%)	5 (1.3%)	7 (1.8%)
Moths		(Lepidoptera OR moth*) NOT butterfl*	210	226	8 (3.8%)	44 (21.0%)	4 (1.9%)	7 (3.3%)
Bats		(Chiroptera OR bat*)	163	153	8 (4.9%)	11 (6.7%)	3 (1.8%)	5 (3.1%)
Flies		(Diptera OR fly OR mosquito* OR midge* OR gnat*) NOT syrphid* NOT hoverfl*	101	68	2 (2.0%)	11 (10.9%)	1 (1.0%)	0 (0.0%)
Beetles		(Coleoptera OR beetle*)	97	89	2 (2.1%)	26 (26.8%)	0 (0.0%)	1 (1.0%)
Mammals (other than bats)		(mammal* OR rodent* OR primate*) NOT Chiroptera NOT bat*	27	27	2 (7.4%)	2 (7.4%)	0 (0.0%)	1 (3.7%)
Ants		(Hymenoptera OR Formicidae OR ant OR ants) NOT Apoidea NOT bee*	20	22	1 (5.0%)	2 (10.0%)	0 (0.0%)	0 (0.0%)
Thrips		(Thysanoptera OR thrips*)	9	11	1 (11.1%)	2 (22.2%)	0 (0.0%)	0 (0.0%)
Orthopterans		(Orthoptera OR cricket* OR weta*)	9	9	0 (0.0%)	1 (11.1%)	0 (0.0%)	0 (0.0%)
Cockroaches		(Blattodea OR cockroach*)	8	7	0 (0.0%)	3 (37.5%)	0 (0.0%)	0 (0.0%)
Reptiles		(reptil* OR Gekkota OR gecko*)	1	2	0 (0.0%)	0 (0.0%)	0 (0.0%)	0 (0.0%)
**Diurnal pollinator taxa**	pollinat* NOT nocturnal NOT night AND…							
Bees		(Hymenoptera OR Apoidea OR bee*) NOT ant	16 269	17 337	1511 (9.3%)	344 (2.1%)	547 (3.4%)	4 (0.0%)
Hoverflies		(Diptera OR syrphid* OR hoverfl*) NOT mosquito* NOT midge* NOT gnat*	1075	1254	145 (13.5%)	39 (3.6%)	32 (3.0%)	0 (0.0%)
Butterflies		(Lepidoptera OR butterfl*) NOT moth*	918	931	93 (10.1%)	16 (1.7%)	60 (6.5%)	0 (0.0%)

Three major groups of diurnal pollinators are included for comparison, showing that nocturnal pollinator taxa are poorly represented in the scientific literature. Searches were conducted using Boolean terminology on 5th May 2020, and the total number of hits recorded in each search engine. Generic search terms for nocturnal and diurnal pollinator taxa respectively were combined with taxon-specific terms to create the exact query for each row (generic and taxon-specific terms are given in columns 2 and 3 respectively). Taxa are ordered within guilds from most to least hits in WoS. Within the hits for each taxon, we conducted additional key-word searches to assess coverage of a range of research topics we have identified for future attention. For each additional search, the number of hits is presented along with the percentage of all hits for the corresponding taxon.

Anthropogenic change to ecosystems has impacted insect diversity and abundance, and insect pollinators in particular are widely considered to be in global decline [8]. This is of substantial concern because over one-third of global crop production by volume depends on animal pollination [9], as well as culturally important or endangered wild plant species [10,11]. Understanding the key ecological, social and economic impacts of current pollinator declines, and the potentially undervalued role of wild pollinator taxa in pollination service provision, are considered key research priorities [1]. Wild pollinators (including wild bees, syrphid and non-syrphid flies, and other taxa) provide the majority of pollination services [12]; despite common assumptions to the contrary, domestic honey bees play only a supporting role [13,14]. Visits from wild pollinators can be as efficient and effective [15], or even more so [16], than those from honey bees, and provide a substantial proportion of all flower visits to a range of crops globally [17]. Evidence exists of community-level declines in insect abundance and biomass [18–20], taxon-level declines in total abundance, biomass, or diversity of beetles, caddisflies, butterflies, moths [21–24], and species-level declines in a wide range of insect taxa (e.g. [25]). Although not all taxa have shown declines [26], change may be regionally [27] and temporally [24] variable. Drivers of these declines are hypothesized to include climate change, habitat loss and fragmentation, agrochemical use, artificial light at night, and changing biotic interactions with pathogens, invasive non-native species, and wild plant resources [8,28–30], with likely interactions between combinations of drivers [31]. However, the relationship between pollination services and wild pollinator diversity is not straightforward [32]. Therefore, it is now of paramount importance to understand the range of wild pollinators that can contribute to pollination services, and the relative importance of each service provider. Among wild pollinators, the nocturnally active guild have been particularly overlooked, largely because of practical obstacles to using standard field methodologies at night-time [33].

Through this review our aim is to promote nocturnal pollination as an area of research that is underrepresented. We illustrate the relevance of nocturnal pollinators by highlighting known nocturnal pollination services, and discuss key mechanisms that underpin interactions between nocturnal pollinators and plants. We highlight evidence which demonstrates how anthropogenic disruptors impact these fragile mechanisms. We conclude with recommendations on where future research would serve to expand knowledge and alleviate threats to nocturnal plant-insect mutualisms.

## Nocturnal pollinators and pollination services

### Moths

**Figure 1.**
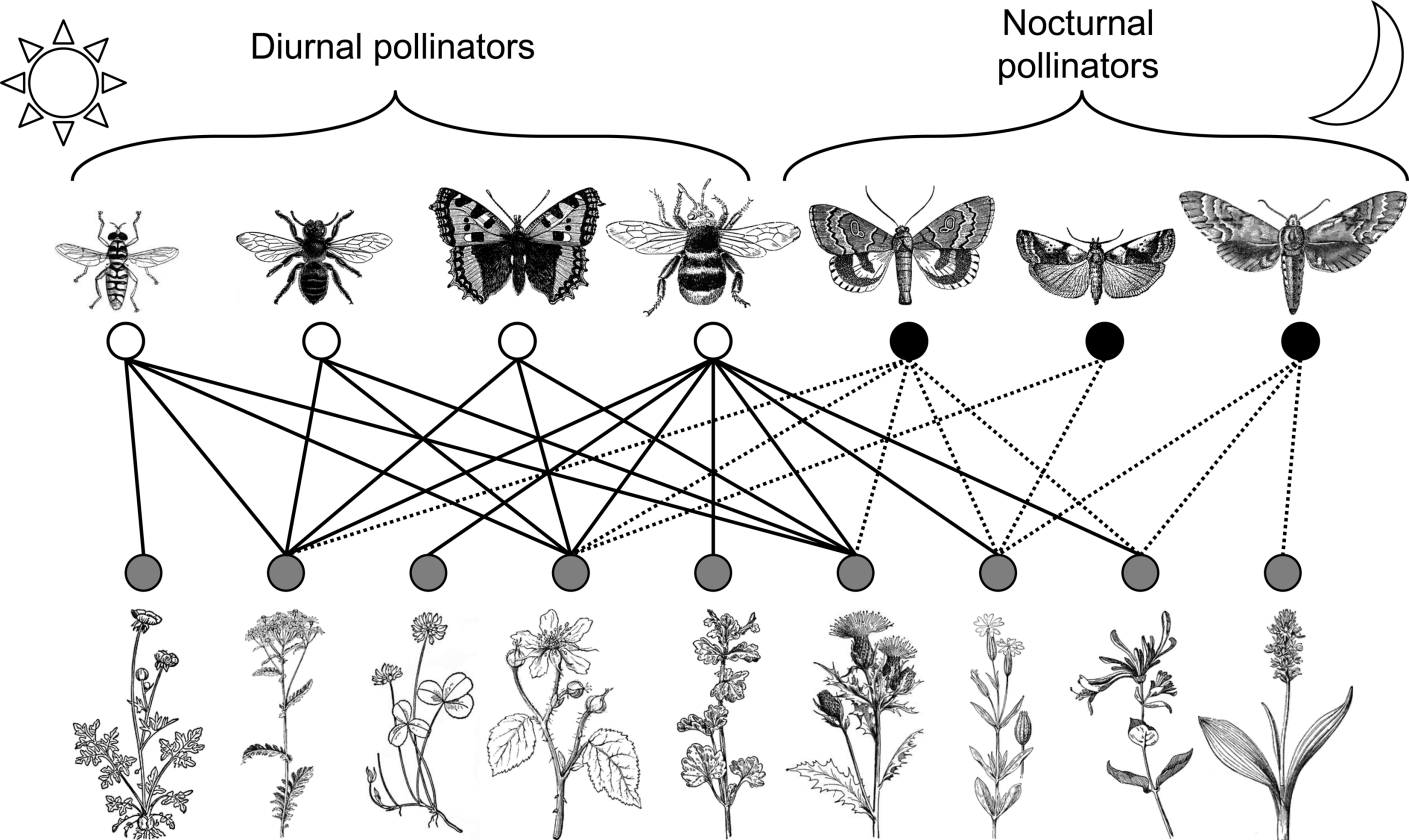
An illustrative temperate grassland network incorporating nocturnal moths. Many moth species are generalist nectarivores as adults, and may provide redundancy to diurnally pollinated plants. Nodes represent species: white = diurnal insects, black = nocturnal insects, grey = plants. Links represent hypothetical pollination interactions: solid = diurnal, dashed = nocturnal. Nocturnal interactions are derived from [33] and diurnal interactions from [133]. Reproduced from [33] under a CC BY 4.0 licence (see [33] for full image credits).

At present, moths (Lepidoptera) probably represent the best-studied of the nocturnal pollinator taxa (Table 1), being of importance in both temperate and tropical zones [33]. A widely held perception is that moth pollination consists primarily of highly specialized, co-evolved interactions between single species of Sphingidae and plants (often Orchidaceae); whilst such interactions do exist (e.g. [34]), this view is not supported by review of the global literature [33], because like butterflies, many moth species are generalist nectarivores as adults (Figure 1). Recent studies have highlighted the richness of moth-flower interactions at community-level [7,35,36] and the potential for moths to interact with, and substantially increase pollination success in, generalist flowers (Figure 1), even in the presence of diurnal pollinators [37].

### Beetles

Beetle (Coleoptera) pollination also includes both examples of specialized flowers featuring beetle-attracting traits [38], and visitation to generalist flowers [39]. Beetles more commonly visit flowers in search of pollen or edible flower parts than nectar [39]. Among beetle-pollinated flowers, nocturnal visitation is most strongly associated with ‘chamber blossoms', whereas other syndromes (especially ‘painted bowls') are visited diurnally [39].

### Bees

Over 250 species from at least four families of bees (Apoidea: Andrenidae, Apidae, Colletidae and Halictidae) have been described with nocturnal or crepuscular activity [40], with multiple hypotheses proposed for why some bees might have evolved to forage at night. Such strategies could reduce competition for nectar and pollen rewards, which are often more abundant in the early morning and late at night [40,41]. Additional advantages, such as avoiding predation and parasitism, may also be a factor [41].

### Bats

Pollination is provided in the tropics by bats (Chiroptera) of the families Pteropodidae and Phyllostomidae, including both specialist nectarivores and opportunistic frugivores [42]. Bat-pollinated flowers are typically large in size and frequently tubular in shape [43]; as such, those that are generalist often have other long-tongued taxa as additional pollinators (e.g. hawk-moths and hummingbirds [44]) rather than generalist bees.

### Thrips

Flower-thrips (Thysanoptera) are minute insects, highly elusive due to their nocturnal and thigmotactic (i.e. crevice-seeking) behaviour. Many species are intrinsically linked to their hosts as brood-site pollinators [45]; thrips exchange pollination for food and reproductive sites in flowers (e.g. [46,47]). They may be particularly effective at pollinating species traditionally thought to be wind-pollinated, with traits such as large numbers of flowers, large quantities of powdery pollen, and large stigmas [48].

### Others

Further to the mounting evidence for pollination service provision by the above taxa, a range of other groups of nocturnal invertebrates and vertebrates have been reported to act as pollinators. Notably, whilst some groups of diurnal Diptera (especially Syrphidae [49] but also others [50]) are well-characterized as pollinators, there has been less attention on flower-visiting species in crepuscular and nocturnal groups; e.g. fungus gnats [51]. Beyond Diptera, recent work has revealed a number of previously unsuspected functional nocturnal pollination interactions between small mammals (including mice and elephant-shrews) and several plant species in South Africa [52,53], suggesting that a considerable amount remains unknown about flower-visiting behaviour even in generally well-studied taxa like mammals. More generally, further night-active taxa including species of cockroaches (Blattodea), grasshoppers and crickets (Orthoptera), ants (Hymenoptera), geckos and primates have all been documented acting as pollinators. Knowledge of the efficiency and effectiveness of these taxa and others as nocturnal pollinators of generalist plants remains extremely limited (Table 1).

### Current evidence for a ‘nocturnal pollination service'

Ecosystem functions become ecosystem services where they provide a tangible direct or indirect benefit to humanity [54]. Whilst it is accepted that pollination is an ecosystem service, the value of nocturnal pollinators is unclear. Nonetheless, evidence supports the existence of at least three classes of nocturnal pollination service: pollination of commercially valuable crops, pollination of culturally important plants, and pollination of rare and endangered species. Beyond these examples of directly observable services, pollination in general underpins effective vascular plant reproduction [55], which in turn is critical to a broad spectrum of further ecosystem functions (and by extension services) [56,57]. However, it is near-impossible to quantify the value of such pollination, or the contribution of nocturnal pollinators towards it.

The clearest example of a nocturnal pollination service is the pollination by bats of a range of commercially important crops in the tropics, most notably *Agave tequilana* F.A.C.Weber (Asparagaceae; the source of tequila) and *Durio* spp. (Malvaceae; durian fruit) [42]. Bats visit flowers of *Durio zibethinus* to feed on pollen shed in late evening, incidentally pollinating the flowers [58]. Durian flowers can be damaged during pollination [58], which is often misconstrued as loss of potential fruit and income, leading to negative attitudes and culling of pteropodid bats as agricultural pests [59]. Thrips are more often considered as pests of temperate crops, yet there are an increasing number of studies which demonstrate their contribution to pollination in crops in tropical and subtropical regions [60], including *Solanum melongena* L. (Solanaceae; aubergine) [46] and *Elaeis guineensis* Jacq. (Arecaceae; oil palm) [61]. Thrips also inhabit flowers of *Coffea canephora* Pierre ex A. Froehner (Rubiaceae; coffee) in high abundance, carrying pollen on their bodies, and may therefore supplement the pollination services of bees [62]. Fewer studies of thrips pollinating temperate crops exist, but *Sambucus nigra* L. (Adoxaceae; elderflower) flowers open at night and fruit set was found to be reduced in populations in southern England when thrips were excluded from inflorescences [47]. A range of Myrtaceae fruit crops are pollinated by nocturnal bees in South America [63]. Moths are also considered possible crop pollinators, though with weaker evidence. Moths have been recorded transporting pollen from several insect-pollinated crops in a UK agro-ecosystem [36], and have also been recorded visiting oil palm [61]. However, it has not yet been established that moths increase the productivity of any commercial crop. To the best of our knowledge, there is only one documented example of nocturnal beetles pollinating a crop species: *Myristica fragrans* Houtt. (Myristicaceae; commercial nutmeg) [64]. Finally, *Persea americana* (Lauraceae; avocado) is primarily considered to be bee-pollinated, but under marginal climate conditions can become night-flowering, when it is visited by a generalist suite of pollinator taxa [65].

Of substantial social and cultural value (but of regional, rather than global commercial value), is *Paullinia cupana* Kunth (Sapindaceae; guarana), a caffeine-rich seed widely consumed in South America. A substantial proportion of flower visits take place nocturnally shortly after anthesis [66], with variation in floral volatiles between day- and night-time that may be adapted to maximize attractiveness to both diurnal and nocturnal bees [67]. Similarly, species of Jasmine *Jasminum* spp. (Oleaceae) and Honeysuckle *Lonicera* spp. (Caprifoliaceae) are of regional importance in south and south-east Asia, with a wide range of cultural uses. Thrips feature amongst a wide range of taxa reported to carry pollen of *Jasminum* flowers [68], whilst *Lonicera* species are adapted to moth-pollination [6]. *Stenocereus queretaroensis* (F.A.C.Weber ex Mathes.) Buxb. (Cactaceae; pitaya), a culturally important crop in central Mexico, displays reductions in yield, fruit quality and seed set in the absence of flower-visits from bats [69].

All groups of nocturnal pollinators provide pollination to wild plants, and amongst these may be ecologically important, rare and endangered species [42]. In addition to generally maintaining the cultural ecosystem services provided by all wild plants, pollination of designated conservation-priority species tangibly benefits humans since investment of time or resources into conservation of such species will be wasted if they cannot reproduce. One such example is *Platanthera bifolia* (L.) Rich. (Orchidaceae; Lesser Butterfly Orchid), the subject of targeted conservation efforts in England [70]. *P. bifolia* is pollination-limited [71] and pollinated by nocturnal moths [72]; therefore its pollinators must play a role in ensuring the project's success. Likewise, studies of the South American tree species *Ocotea porosa* (Nees & Mart.) Barroso (Lauraceae), listed as Vulnerable on the IUCN Red List, suggest it is primarily or exclusively pollinated by the thrips *Frankliniella gardeniae* (Moulton, 1948), leading to calls for thrips to be explicitly considered in conservation planning for this species [73]. At a broader scale, 31% of cactus species (Cactaceae) are assigned to IUCN Red List categories Vulnerable, Endangered or Critically Endangered [74]. Since many cacti (especially ceroid cacti) are adapted to pollination by bats and/or moths [33,42], a nocturnal pollination service may apply to any efforts to conserve these species.

Whilst we have acknowledged, in this section, the full taxonomic breadth of providers of nocturnal pollination (including the often-important role of bats as providers of nocturnal pollination), we will henceforth remain within the authors’ areas of particular expertize, referring primarily to insect pollinators, and especially to moths and thrips.

## Key mechanisms for night-time pollination interactions

Critical to pollination are the individual-level interactions between plants and their pollen-vectors. From the pollinators’ perspective, the opportunity to obtain a food reward (or occasionally to reproduce) will drive these time-critical interactions. Plants must have mechanisms to attract an effective pollinator and in turn, pollinators must efficiently detect and decipher plant cues to compete for potentially limited resources. Here we describe some of the mechanisms that are important in underpinning these interactions in nocturnal pollination systems.

### Floral scent

Floral scent is important in many nocturnal plant-pollinator mutualisms [75]. Floral volatile chemistry has been characterized for taxa pollinated by moths [76–78], beetles [38,79,80], nocturnal bees [63,67,81], and to a lesser extent smaller nocturnal and crepuscular pollinators, such as mosquitoes [82] and thrips [47,83]. Parallels can be drawn between floral scent profiles of plants associated with nocturnal pollinator taxa; for example, moth-pollinated flowers often emit a combination of acyclic terpene alcohols (e.g. linalool), aromatic alcohols (e.g. benzenoids), derived esters, and trace nitrogen-containing components [76–78]. Some compounds are relevant to the attraction of multiple nocturnal taxa, such as linalool, which may attract moths, bees, mosquitoes, thrips and others [47,82,84,85]. However, this may only become apparent when the timing of the release of component volatiles in scent emissions is quantified, since floral scent emissions are often rhythmic [67,86]; for example, in *Petunia* spp. (Solanaceae) pollinated by hawk-moths (Sphingidae), diurnal volatile emissions are significantly less attractive to pollinators than nocturnal emissions [87]. Such rhythmic floral scent emissions, driven by an internal gene-regulated circadian clock [3], are often most evident in long-lived flowers associated with nocturnal pollinators [47,86]. Emissions of volatiles in circadian rhythms can be an adaptation to nocturnal pollinator behaviour [88], but avoiding attraction of (or repelling) diurnal herbivorous insects [89] can equally contribute to adaptive selection of nocturnal scent emissions in some species.

### Night vision

Nocturnal insects have extraordinarily advanced visual systems, including scotopic colour vision [90], which enable them to navigate within and between flowering plants. Nocturnal compound eyes are highly adapted to obtain enough light to generate optical stimuli, whilst limiting internal physiological noise which threatens the clarity of visual signals [40]. Fully nocturnal taxa (e.g. moths) typically have superposition compound eyes, with complex adaptations to improve sensitivity [40], but some nocturnal Hymenoptera have retained and adapted the apposition eyes of their day-active relatives [91,92]. Common adaptations required for either nocturnal eye form lie in the ability to slow the processing response of photoreceptors and manage the summation of the light signals in space and time, enabling insects to maximize light and movement sensitivity in low light conditions. Nonetheless, it is most probable that nocturnal pollinators combine vision with other senses when foraging. This is evident in crepuscular bees that fly exclusively in dim light conditions (e.g. *Ptiloglossa*: Colletidae and *Megalopta*: Halictidae), using a combination of visual [93] and olfactory senses to seek floral rewards [94]. From the plants’ perspective, many species adapted to nocturnal pollination (especially by moths) are pale or white in colour, likely to increase visibility in low light conditions [95].

### Thermogenic sensitivity

In addition to reduced light, cooler ambient temperatures are characteristic of the nocturnal environment. Most pollinating insects are ectotherms, so the ability to distinguish flowers offering floral warmth would be advantageous [75]. Flowers can retain external heat [96] or produce their own [97], and plants producing heat rewards, especially those growing in in cooler environments, may be preferentially visited by ectothermic pollinators [98,99]. In addition to providing warm shelter, thermogenesis increases floral scent volatilization [100]. Thermogenesis occurs widely among both gymnosperms and angiosperms, including species of cycads (e.g. Zamiaceae) pollinated by thrips [101] and beetles [102], and species of Araceae pollinated by beetles and flies [103,104]. While it is difficult to isolate heat stimulus from other stimuli to afford importance of floral heating as a pollinator attraction mechanism, it has been proposed that nectar-feeding insects with the capacity to detect infrared (IR) thermal radiation (e.g. mosquitoes and other blood-feeding taxa) could use this to detect to detect and track the floral nectaries of thermogenic flowers at night [105]. Certainly, plants appear to exploit pre-existing stimuli in other, similar ways in order to attract their pollinators (e.g. by producing volatiles that are chemically similar to pheromones [38]), but nocturnal thermogenic attraction has not yet been demonstrated to occur.

## Anthropogenic disturbance of nocturnal pollination

The reliance of nocturnal pollination systems on such complex interspecific plant-insect signalling mechanisms means that they are highly vulnerable to influence by their unique abiotic and biotic environments. Anthropogenic drivers of environmental change, especially large-scale habitat loss and global climate change, are implicated in driving pollinator declines [106], with severe implications for insect-pollinated plants [29]. However, an insidious effect of such drivers is their potential to directly disrupt the fine balance of mutualisms between plants and pollinators at a local scale. Such effects are harder to detect than declining pollinator abundance or distribution, but may have equivalent impacts on plant reproductive success by reducing effective visitation rates. Here we discuss three anthropogenic drivers of change with the potential to disrupt each of the key mechanisms described above.

### Pollution

Environmental pollution takes many forms, and can in turn disrupt scent-based communication between flowers and pollinators through several mechanisms. Most obviously, air pollution (release of anthropogenic volatile pollutants (AVPs) from traffic, industry etc.) provides direct interference to such communication. AVPs simply add background noise to floral signals, masking their detection [107], but also increase degradation rates for floral volatiles [108,109] including linalool [110], reducing signalling efficiency and range. Less directly, nitrogen enrichment of habitats (linked both to air pollution and agricultural intensification [111], and considered a threat to biodiversity e.g. [112]) may also influence floral scent. Soil nitrogen availability influences production and emission of a range of plant volatiles, including those involved in plant-insect signalling [113], though evidence of such effects on floral volatiles specifically is limited [114,115]. It is plausible that changes in other properties of soil chemistry might have similar effects [116], but this does not appear to have been extensively studied.

### Artificial light at night

Artificial light at night (ALAN) is increasingly understood to be an important and increasing source of ecological disruption [117], and may impact both visual and floral scent cues for nocturnal pollinators. Presence of bright sources of ALAN can lead to rapid reductions in ocular sensitivity, inhibiting night-time vision away from the light source [33]. Such light sources may also alter the nocturnal colour environment, with the result that some flowers stand out more from their environment and others are masked, compared with natural night-time light spectra [33,118]. Such effects might benefit certain plant species through increased visitation rates, but unbalance plant-pollinator interactions at the community level [118]. Regarding floral scent, adaptive circadian rhythms are important in timing the release of floral volatiles in some nocturnally pollinated plants [119], and these are likely to be disrupted by presence of ALAN obscuring photoperiodic cues [120]. If this were to result in reductions in visitation by preferred pollinator taxa [37], and/or increase visitation by less efficient taxa, then there may be a possibility of reduced productivity and higher floral costs, but such effects have not been investigated. Finally, ALAN is understood to directly disrupt pollinator behaviour, with the likely outcome that less time is spent foraging (and therefore fewer flowers visited). For example, in moths, exposure to ALAN reduces feeding behaviour [121] and increases flight activity at the height of street-lamps, away from flowers [122].

### Climate change

The general effects of elevated global temperature on plant-pollinator interactions are diverse, and are reviewed in detail elsewhere in this issue [123]. With specific regard to thermogenic sensitivity and its hypothesized role in detection of flowers by certain pollinators, two factors may determine the impacts of climate change on such interactions. First, thermogenic plants must be able to maintain thermogenesis under elevated temperatures. Given that thermogenesis operates by increasing respiration rates at higher ambient temperatures [97], climate warming may lead to physiological limits to respiration rates being encountered, preventing the continuing maintenance of thermogenesis [124] and altering the efficiency of thermogenic sensitivity detection as a flower-visitation mechanism [99]. Second, pollinator preference may itself change, with thermogenic flowers becoming undesirable if ambient temperatures increase enough to place pollinators under thermal stress [125]. Thus, insects using infrared radiation to seek out thermogenic flowers may rely less on this cue to seek floral resources under elevated temperatures (or even use it for avoidance), potentially negatively impacting plant-pollinator interactions at community level as described for ALAN.

## Discussion

In this review we have highlighted the diversity of nocturnal pollinators and the complexity of their interactions with plants, but it is clear that much remains unknown about the extent, value and vulnerability of pollination services that occur at night. Evidence of pollination services provided by nocturnal foragers to economically important crops is becoming increasingly apparent [36,47,65]; nonetheless, such services have been much less extensively studied than for diurnal pollinators (Table 1). The visual and olfactory systems of nocturnal pollinator taxa, and the corresponding signalling mechanisms of plants, are reasonably well-studied (Table 1), but often from a single-discipline perspective (plant or insect) and consequently large knowledge gaps remain. Importantly, all specialized nocturnal detection and signalling mechanisms are liable to be directly or indirectly disrupted by anthropogenic environmental change (including air pollution, changes in soil chemistry, artificial light at night, and climate change), with potential detrimental effects for pollination services which have not been quantified. Increased understanding of the functioning of nocturnal pollination systems is critical for future efforts to conserve their ecological service. To this end, we have identified a number of important directions for future research.

### Deeper understanding of nocturnal pollination services

Given the vital importance of effective pollination to the yields of many crops, the major pollinators of such crops are likely to be well-characterized in most cases. It seems unlikely, therefore, that there are any remaining unknown examples of commercially-important crops where the dominant pollinator comes from a nocturnal guild. However, the challenges of nocturnal activity, especially when combined with high mobility, minute size or cryptic habits can confound study of night-time flower visitors, and this may have contributed to the low proportion of the nocturnal pollinator literature addressing ecosystem service provision (Table 1). Considering that diurnal pollination systems are widely generalized in nature even when individuals (or even species) act as specialists [5], nocturnal pollination systems may be more generalized than previously assumed (especially for moths [35]), so nocturnal pollinators may provide previously unassessed supplementary pollination to generalist crops (e.g. [65]), which remains to be evaluated. In contrast, the pollination systems of rare and endangered plant species may not necessarily be well-characterized. Establishing the full range of taxa which pollinate a given species, and their relative importance, should therefore form an important component of any plant conservation effort. Besides simply adding redundancy to pollination systems, nocturnal pollination could bring further benefits to generalist plants which also warrant investigation. For example, nocturnal moths have been shown to disperse pollen over greater distances than diurnal pollinators in several plant species [6,126,127], potentially increasing gene flow and maintaining genetic diversity within and among populations.

Particularly important to quantifying nocturnal pollination services will be deeper knowledge of the trade-off between services and disservices when nocturnal pollinators (including moths, thrips and beetles) are also herbivorous, either at the same life stage [47] or as larvae [128]. At present this trade-off is extremely poorly understood except in a few specialized cases (e.g. [129]), but misunderstanding of such trade-offs can lead to deleterious persecution of beneficial mutualists [59]. Likewise, there is virtually no existing knowledge about how this balance between ecosystem services and disservices may be affected by the many anthropogenic drivers of environmental change that can impact moths, thrips, beetles and bats; e.g. if disruption of floral scent rhythms were to reduce both attraction of nocturnal pollinators and repellence of diurnal herbivores. Increasing our understanding of these trade-offs and how they may be changing is important, but must also be viewed with a degree of nuance, since there is likely to be a high degree of variation in these impacts between different systems and contexts.

### Improved mechanistic understanding of individual nocturnal insect-flower interactions

Visual and olfactory systems, and their relationship to flower-visiting activity, are well understood in some taxa (e.g. moths [130]) but not in others [75], so a broader mechanistic understanding would be valuable. In particular, the relative importance of different mechanisms in low-light conditions at night is not known, and is likely to differ from diurnal pollination systems. More specifically, the hypothesized role of thermogenic sensitivity in attracting pollinators of certain taxa has not been established at all, and it is not even known whether heat rewards play a role in nocturnal systems, as has been shown for diurnal bees [99], despite the fact that heat rewards could be especially valuable to insects foraging in cooler night-time temperatures. From the plants’ perspective, a greater understanding of the differences between floral signals in diurnal and nocturnal species might elucidate the selective pressures that have driven the repeated convergent evolution of nocturnal flowering strategies.

### Impacts of environmental change on individual nocturnal insect-flower interactions

Whilst we have described plausible mechanisms by which nocturnal pollination services may be disrupted by a range of anthropogenic drivers of environmental change, little research currently exists to demonstrate the existence of such effects, or their importance/value. Climate change alone forms a greater proportion of the diurnal pollinator literature than climate change and ALAN combined in studies of nocturnal pollinators, despite the importance of ALAN to nocturnal systems (Table 1). Furthermore, much of the existing research investigating environmental factors which influence floral scent composition has been focused on diurnal pollination systems, even though studies of floral scent form a relatively large section of the nocturnal pollinator literature (Table 1). If abundance and composition of floral scent is of greater importance at night, due to low-light conditions reducing the utility of visual cues, then the impact of any environmental fluctuations on nocturnal plant-pollinator systems may be more extreme. Although better-studied than climate change, the effects of artificial light at night on nocturnal pollination may vary from species to species [33,37,118,122], making it challenging to explicitly predict impacts on nocturnal pollination services. Species-by-species assessments may therefore be necessary, and could ultimately allow traits determining whether effects of ALAN are beneficial or deleterious to be identified. The effects of other drivers, and on other plant-insect communication mechanisms, are almost entirely unstudied, but could underlie surprisingly large impacts on pollination systems that will be important to identify [99].

### Conclusions

At a time when public and scientific concern about insect declines has never been greater [131], improving our understanding of the scale and value of nocturnal pollination services (to crops and wild plants of conservation concern) may be a powerful tool to advocate pollinator conservation [132], including of a range of non-bee taxa (but see [32]). This may be especially important for taxa that are often viewed as agricultural pests, but which may simultaneously be underappreciated ecosystem service providers. However, it is not merely the insects themselves that are under threat from a range of anthropogenic drivers of environmental change: the very mechanisms by which plants and their pollinators communicate are vulnerable to disruption. Understanding the nature of such disruptions is therefore of vital importance in order to develop effective mitigation strategies and ultimately conserve the nocturnal pollination service.

## Summary

- Understanding the importance of entire guilds of pollinators, including nocturnal taxa, is vitally important at a time of public concern over biodiversity declines.
- Pollination services are provided nocturnally by a wide range of taxa including moths, bats, bees, thrips and beetles.
- Nocturnal interactions between plants and pollinators are maintained by complex, specialized mechanisms, but these mechanisms can be disrupted by anthropogenic drivers of environmental change.
- Improved understanding of the scale of nocturnal pollination, and the potential disruption caused by environmental change (including the possibility that disruptions shift the status of the relationship from beneficial to harmful), will be critical to ensuring pollination services are conserved in the future.

## Open Access

Open access for this article was enabled by the participation of University of York in an all-inclusive *Read & Publish* pilot with Portland Press and the Biochemical Society under a transformative agreement with JISC.

## Abbreviations

ALAN: Artificial light at night
AVPs: anthropogenic volatile pollutants
